# Solitary flexural–gravity waves in three dimensions

**DOI:** 10.1098/rsta.2017.0345

**Published:** 2018-08-20

**Authors:** Olga Trichtchenko, Emilian I. Părău, Jean-Marc Vanden-Broeck, Paul Milewski

**Affiliations:** 1 Department of Physics and Astronomy, University of Western Ontario, London, Ontario, Canada; 2 School of Mathematics, University of East Anglia, Norwich, UK; 3 Department of Mathematics, University College London, Gower Street, London, UK; 4 Department of Mathematical Sciences, University of Bath, Claverton Down, Bath, UK

**Keywords:** solitary waves, flexural–gravity waves, boundary integral method

## Abstract

The focus of this work is on three-dimensional nonlinear flexural–gravity waves, propagating at the interface between a fluid and an ice sheet. The ice sheet is modelled using the special Cosserat theory of hyperelastic shells satisfying Kirchhoff's hypothesis, presented in (Plotnikov & Toland. 2011 *Phil. Trans. R. Soc. A* **369**, 2942–2956 (doi:10.1098/rsta.2011.0104)). The fluid is assumed inviscid and incompressible, and the flow irrotational. A numerical method based on boundary integral equation techniques is used to compute solitary waves and forced waves to Euler's equations.

This article is part of the theme issue ‘Modelling of sea-ice phenomena’.

## 1. Introduction

In this paper, we consider an incompressible and inviscid fluid covered by an ice sheet. Under certain conditions, the floating ice sheets can be modelled as an elastic medium, and their interaction with the fluid makes the resulting hydroelastic problem mathematically challenging [1]. A renewed interest in waves generated by moving loads on top of floating ice sheets has been sparked in the last 40 years by a series of experiments in cold regions (e.g. [2–6]), where ice roads and runways are used during the winter. More recently, the displacement of ice cover generated by moving vehicles has been measured using radar satellites [7]. Waves under ice cover can be generated by other events, such as a landslide-generated tsunami [8].

There are a variety of models for an ice sheet floating on top of a body of water, starting with the linear model used by Greenhill [9] or the Kirchhoff–Love plate model [10], another popular model used in past decades. In this work, the model for the ice sheet uses the special Cosserat theory for thin hyperelastic shells, satisfying Kirchhoff's hypothesis, described in detail in [11].

Most of the analytical and numerical studies of nonlinear flexural–gravity waves (or hydroelastic waves) concentrate on two-dimensional problems. Weakly nonlinear models, such as the nonlinear Schrödinger equation or the fifth-order Korteweg–de Vries equation, have been derived to analyse flexural–gravity waves [12,13]. The existence of solitary flexural–gravity waves has been studied using central manifold theory [14,15] or variational techniques [16]. Also, the existence of travelling flexural–gravity waves was proved using critical points of a Lagrangian [17], while the well-posedness of the initial-value problem was investigated using the vortex-sheet method [18].

Steady and unsteady solitary waves have been investigated numerically in a variety of configurations by a number of authors, using boundary integral methods and high-order spectral methods [19,20], using conformal mapping techniques [21–23]. Travelling waves and generalized solitary waves have also been computed using different numerical methods such as the Galerkin-type method [10], series expansions [24] or conformal mapping techniques [25] and other non-local methods [26,27].

In most cases, the elastic shell is assumed to be without mass, but very recently theoretical and numerical works have been published that consider the case of heavy hydroelastic waves [28–30]. Other related two-dimensional problems include the study of internal waves under an ice sheet [31], the hydraulic falls under an elastic sheet [32] and the wave attenuation of solitary waves in a fragmented ice sheet [33].

There are fewer three-dimensional studies of flexural–gravity waves, owing to the complexity of the problem. Linearized problem patterns of flexural–gravity waves generated by moving loads have been presented by Davys *et al.* [34] by investigating the dispersion relation. Linear deflections generated by a rectangular load on an ice sheet have been computed using Fourier transforms [35].

Solitary waves in three dimensions have also been studied by deriving a Benney–Roskes–Davey–Stewartson model for a fluid of arbitrary depth covered by an elastic sheet and considering small-amplitude waves [36]. This model predicts that in an infinite depth case there are no small-amplitude solitary waves, but they exist for shallow water. In shallow water, a three-dimensional generalization of the fifth-order Korteweg–de Vries equation was derived [37], which admits solitary waves as solutions. More recently, fully localized three-dimensional solitary waves have been computed in a quintic Hamiltonian model derived from the full nonlinear Euler equations [38].

The focus of this work is on waves generated by moving pressures and on fully localized solitary flexural–gravity waves. These are computed in three dimensions for water of finite or infinite depth, using the nonlinear model formulated in Plotnikov & Toland [11] for the ice sheet. Recently, a Hamiltonian reformulation of the governing equations was presented [39]. The boundary integral method used to perform the computations was previously derived for gravity waves [40,41] and later extended for gravity–capillary waves [42,43]. Waves generated by a moving load on a nonlinear fluid–linear elastic plate configuration have been computed using a boundary integral method [44] and solitary waves have been found [45]. Very recently, the method was modified to compute small-amplitude solutions for Kelvin-wake patterns using Krylov methods, with a preconditioner based on the linearization [46].

The layout of this paper is follows. The next section includes a description of the problem and its reformulation, with the following section describing the numerical method used. The numerical results section follows, including sample wave profiles. The paper ends with conclusions and discussions of the results.

## 2. Formulation

**Figure 1.**
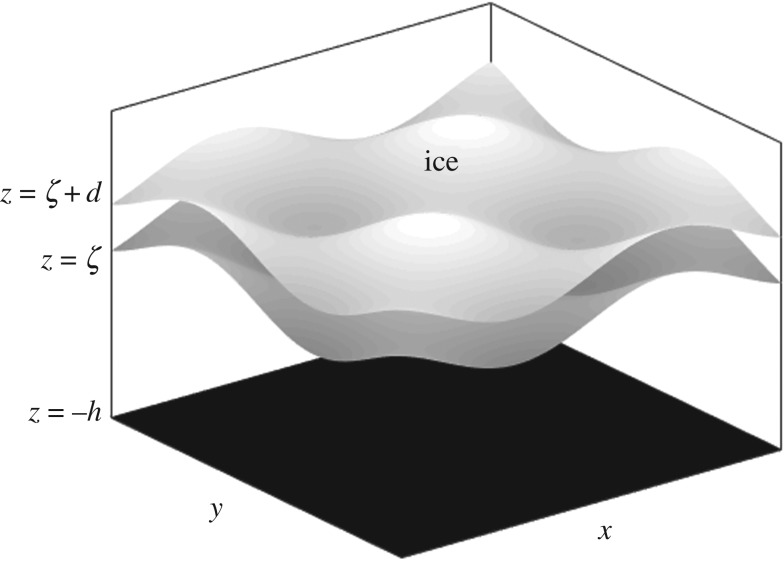
A schematic representing the domain for (2.1).

The Euler equations describing an incompressible, inviscid, irrotational fluid with velocity potential *Φ*(*x*, *y*, *z*, *t*) and variable surface *ζ*(*x*, *y*, *t*) under a sheet of ice in three dimensions are given by
2.1
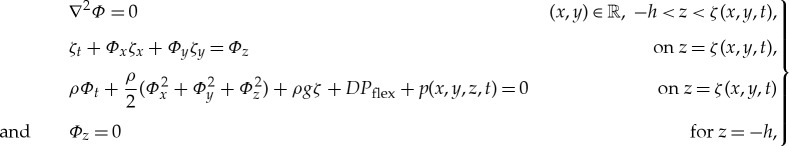
where *ρ* is the density of the water, *D* is the flexural rigidity of ice, *g* is gravitational acceleration, *h* is the depth of the water (which could be taken to be infinite), *p*(*x*, *y*, *z*, *t*) is an external pressure exerted on the ice and *P*_flex_ is a term describing the effect of the ice on the surface of water. The schematic for the domain of interest is shown in figure 1, where the Cartesian coordinates *Oxyz* are defined such that the *z*-axis is oriented vertically upward and the waves propagate along the *x*-axis.

The term describing the effects due to the presence of ice is modelled using the Cosserat theory of hyperelastic shells [11]. It assumes that the ice is a thin elastic plate with constant thickness, the ice bends with the water waves and it cannot break. The model neglects friction between the ice and the water. The coefficient for flexural rigidity for ice *D* is given by

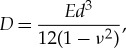
where *E* is Young's modulus (i.e. the modulus of elasticity), *ν* is the Poisson ratio describing the effects of transverse strain relative to axial strain and *d* is thickness of the ice.

After some algebra, we can express *P*_flex_ in Cartesian coordinates as
2.2
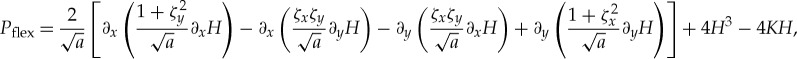
where *H* is the mean curvature and *K* is the Gauss curvature of the ice–water interface, given by

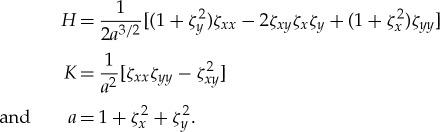
It is worth noting that in previous works on three-dimensional flexural–gravity waves [44,45] a linear model was used for the ice sheet, where *P*_flex_ has a much simpler form as

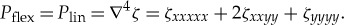


We analyse the linearization of the nonlinear system (2.1) and look for plane wave solutions of the form e^i(*k*_1_*x*+*k*_2_*y*−*ωt*)^ and 
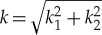
. The linear dispersion relationship between the frequency *ω* and the wavenumbers *k*_1_ and *k*_2_ is then
2.3
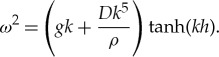
If we only consider two-dimensional waves moving in the *x*-direction with wavenumber *k*, then we can further define a phase speed
2.4
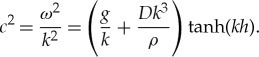
It can be shown that *c* always has a minimum *c*_min_ at a finite wavenumber *k* = *k*_min_ > 0 for all the values of the physical parameters. At this critical speed, the group and phase speeds are equal. For 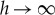, the minimum phase speed *c*_min_ is
2.5
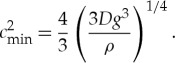


**Figure 2.**
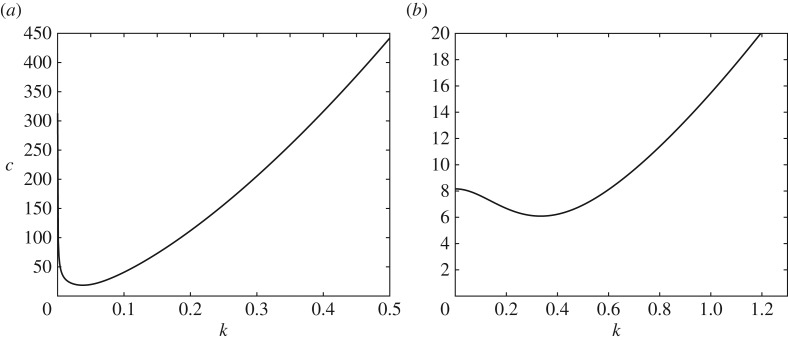
Plots of the phase speed *c*(*k*) in (2.4) using the physical parameters from the experiments described in [6] ((*a*) infinite depth, *D* = 1.6 × 10^9^ Nm) and in [3] ((*b*) finite depth, *h* = 6.8 m, *D* = 2.35 × 10^5^ Nm).

There are very different wave patterns expected when we consider a pressure travelling at a speed *U* above or below this critical speed *c*_min_ from (2.5). In figure 2*a*, we show (2.4) for infinite depth and in figure 2*b* we show (2.4) for finite depth. For *U* < *c*_min_, the disturbance due to the moving pressure approaches a uniform flow at infinity as there is no *k* for which *U* = *c*(*k*), hence no waves are generated in the far field. However, for *U* > *c*_min_, there are two wavenumbers for which *U* = *c*(*k*) and a more complicated pattern emerges. This analysis generalizes to three dimensions and, in this work, we focus on the case of *U* < *c*_min_ and we will look for symmetric solutions in *x* and *y*.

**Figure 3.**
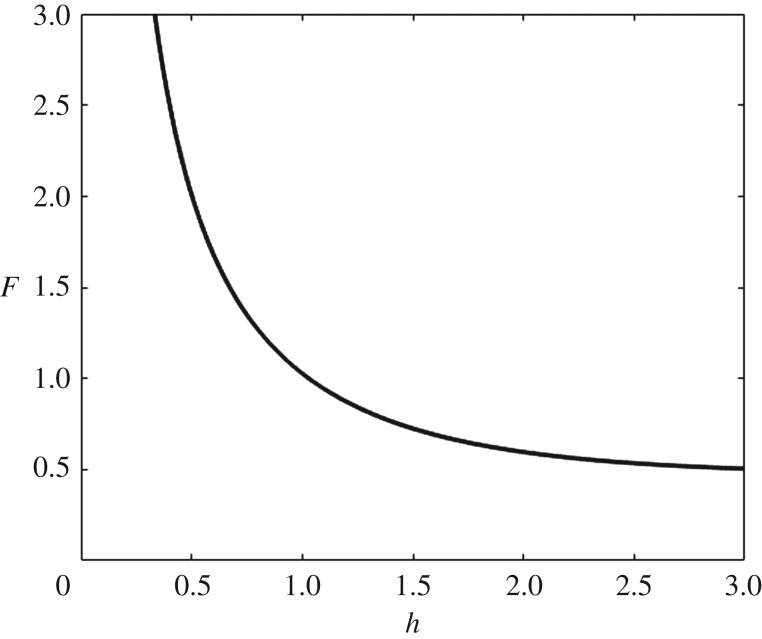
Critical values of *F* versus *h* from which solitary branches of solutions are expected to bifurcate.

Since we restrict our focus to solitary and forced waves, travelling with speed *U*, it is convenient to use a reference frame moving with the wave by setting 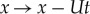 and considering steady-state solutions. We non-dimensionalize the problem using this speed *U* as the unit of velocity and introduce a unit of length *L*. This allows us to rewrite the Bernoulli condition at the surface (the third equation in (2.1)) in terms of the non-dimensional parameters, using
2.6
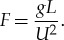
We introduce *β* = *D*/*ρU*^2^*L*^3^ and for convenience 
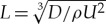
, which will set *β* = 1. The non-dimensional depth is defined as 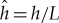, dropping the hat for ease from now on. As mentioned above, there is always a minimum of the dispersion relation, whatever the values of physical parameters. In figure 3, we plot the curve in the *F*–*h* plane, which corresponds to this minimum.

To solve equations (2.1), we use a boundary integral equations method (e.g. [40,42–45]), using Green's functions. We now review the main aspects of the numerical scheme. We note that, for Laplace's equation (the first equation in (2.1)) in three-dimensional free space, Green's function in infinite depth for the points *P* = (*x*, *y*, *z*) and P* = (x*, y*, z*) is
2.7
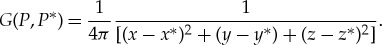
We use Green's second identity, which states
2.8
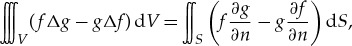
where we can set *g* = *G*(*P*, P*) and *f* = *Φ* − *x*, which satisfies Laplace's equation. After the proper substitutions and some manipulation, we obtain
2.9
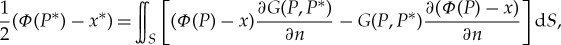
where *n* is the normal to the ice–water interface *S* pointing into the fluid, and P* is a point from *S*.

We define
2.10
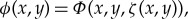
which allows us to rewrite the Laplace equation as well as some of the boundary conditions in terms of a surface integral.

The final form of equations to solve for flexural–gravity waves in infinite depth is
2.11
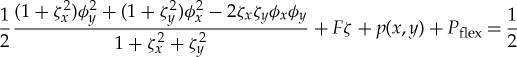
and
2.12
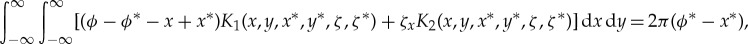
where
2.13
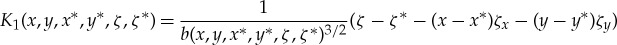
and
2.14
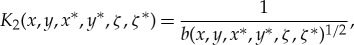
with

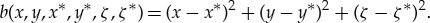
We use the notation *ζ* = *ζ*(*x*, *y*), ζ* = *ζ*(x*, y*) and ϕ* = *ϕ*(x*, y*).

Symmetry in the *y*-direction with *ζ*(*x*, *y*) = *ζ*(*x*, − *y*) and *ϕ*(*x*, *y*) = *ϕ*(*x*, − *y*) implies that we can solve the set of equations (2.11) and (2.12) on half of the domain. We use the method of images to account for the symmetry, which introduces two extra terms in each kernel

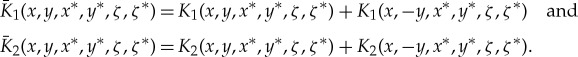
Part of the integral in (2.12) is singular [10] and we remove the singularity by noting that
2.15
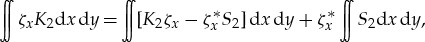
where
2.16
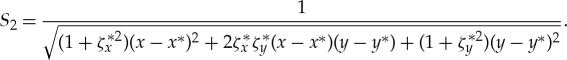
The last term in (2.15) can be computed analytically since it is of the form 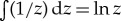.

Moreover, if we consider a fully symmetric solution in *x* and *y*, then

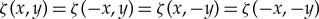
and

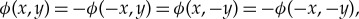
this additional symmetry in *x* will also introduce two more terms to each kernel. This implies that, using Green's function approach, we will have four terms in each kernel due to the method of images. We can thus reduce the computational cost by only integrating over a quarter of the domain. For water of finite depth *h*, we also need to account for the image across the bottom at *z* = − *h* (for more details on the approach for finite depth, see [42]). Thus, the equation (2.12) for water of finite depth becomes
2.17
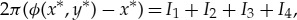
where
2.18
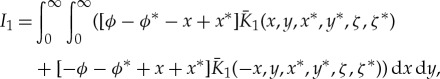

2.19
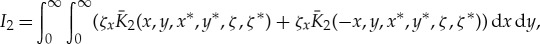

2.20
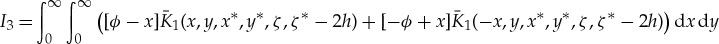

2.21
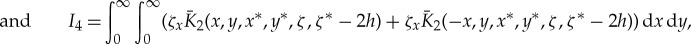
where, as before, *I*_2_ is singular.

## 3. Numerical method

The equations given by (2.11) and (2.17) with the integrals shown in (2.18)–(2.21) are discretized by setting *x*_*i*_ and *y*_*j*_ to be equally spaced points such that *i* = 1, …, *N* and *j* = 1, …, *M*. Let the vector of unknowns be *ϕ*_*x*__(*i*,*j*)_ and *ζ*_*x*__(*i*,*j*)_ such that
3.1
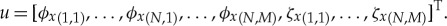
We evaluate the equations at points (*x*_*i*+1/2_, *y*_*j*_) and use finite differences for the derivatives. This gives 2(*N* − 2)*M* equations. We obtain 2*M* equations from symmetry about the *y*-axis and 2*M* more equations from decay at the boundaries. To obtain *ζ* and *ϕ*, we use a trapezoid rule. Derivatives are computed using central difference, second-order accurate schemes, except at the border where one-sided schemes are needed. This gives 2*NM* equations, which can be written as
3.2
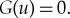


To solve the system, we use Newton's method [46,47], which is summarized as

(i) set up an initial guess *u*^0^
(ii) until convergence
  (a) solve *J*(*u*^*n*^)*δ*^*n*^ =  − *G*(*u*^*n*^)
  (b) set *u*^*n*+1^ = *u*^*n*^ + *δ*^*n*^
  (c) test for convergence and repeat steps if not converged.

This method relies on an initial guess *u*^0^ and the Jacobian matrix *J*. To compute non-trivial solitary waves (ones that are non-zero), we need a good initial guess in order for the numerical method to converge. However, since the set of equations is complicated and very nonlinear, there is no analytical expression for it. In order to obtain solitary waves, the numerical procedure is to compute a branch of forced waves, reduce the forcing, and use that as an initial guess. The particular form of the forcing pressure is not important. The main requirement is that it has a bounded support. Here we choose
3.3
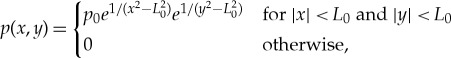
and, for convenience, we choose *L*_0_ = 1 and *p*_0_ to be small, positive or negative. To obtain fully nonlinear waves, we use a continuation method. We start with a small amplitude, using the flat water as an initial guess with *F* > *F*_min_, where *F*_min_ corresponds to the minimum of (2.4). We use Newton's method to compute the true solution for the chosen *F*, and then rescale to use as an initial guess for a larger amplitude solution. This process is continued until we go around a turning point in the bifurcation branch for the forced solutions. Then, decreasing *p*_0_, we can compute non-trivial solutions to the unforced (free surface with *p*_0_ = 0) problem. We expect bifurcation of solitary waves to start from a point on the curve shown in figure 3 corresponding to the depth *h* considered, and solitary waves to exist for *F* > *F*_min_. Similar approaches were used before for gravity–capillary waves for the two-dimensional problem [48] and for the three-dimensional problem [42,43].

Another key component to Newton's method is the Jacobian matrix *J*. Owing to the form of the equations we are solving, (2.11) and (2.17), this Jacobian is hard to compute analytically. Therefore, it is computed numerically via finite differences. For a three-dimensional problem, this matrix can be quite large and it requires a lot of memory to store and also to compute its inverse. In particular, due to the high degree of the nonlinearity from the flexural term (2.2) and the presence of the double integral over the whole space, this Jacobian matrix contains at least two dense quadrants. In general, there are two ways to reduce the computational requirements of Newton's method. One way is to compute an approximate inverse of the Jacobian and another way is to compute an approximate Jacobian [47]. It is known that iterative solvers such as GMRES can compute approximate inverses, but these require preconditioners to converge well. For this problem, preconditioners can be found from previous steps along the bifurcation branch; however, they require a lot of memory to store so this process is inefficient. It is faster to not update the Jacobian at every step along the bifurcation branch, hence we use only an approximate Jacobian. However, for this flexural–gravity wave problem with the nonlinear term due to the Cosserat model used, having an inexact Jacobian was found to result in poor convergence.

## 4. Numerical results

**Figure 4.**
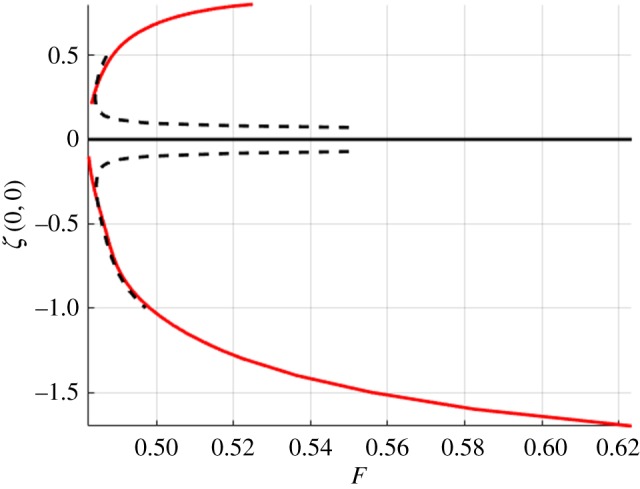
Bifurcation branches for forced (dashed) and solitary (solid) waves in infinite depth. (Online version in colour.)

**Figure 5.**
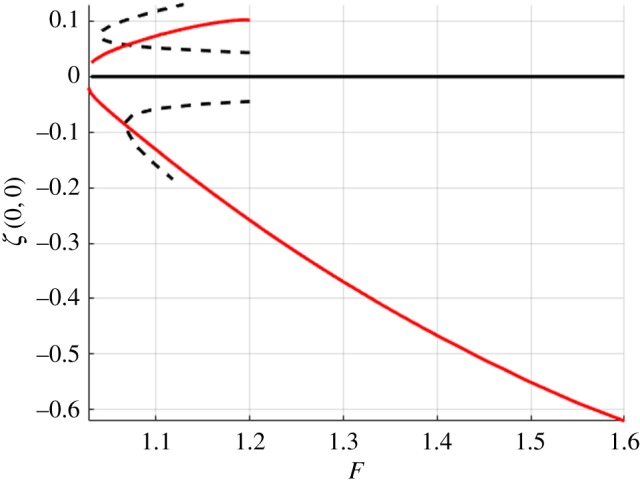
Bifurcation branches for forced (dashed) and solitary (solid) waves in finite depth with *h* = 1. (Online version in colour.)

In this section, we present the numerical results for forced and free surface waves. For what follows, we use the doubly symmetric equations on a quarter of the domain with *n* = 80, *m* = 50, Δ*x* = 0.5, Δ*y* = 0.8. The accuracy of the method was checked by varying Δ*x* and Δ*y* and the number of points in each direction. The tolerance for convergence of Newton's method was set to 10^−10^. The forcing term (3.3) had *p*_0_ = 1 for depression waves and *p*_0_ = − 1 for elevation waves (unforced *p*_0_ = 0). Figures 4 and 5 summarize the computations using a continuation method with a Newton iteration at every step. These figures show the bifurcation branches for both depression and elevation waves with and without a forcing. Dashed lines show the forced waves, which were used as a starting point for the computations of free solitary waves and computed for *U* < *c*_min_, and solid lines are the solitary waves without a forcing.

**Figure 6.**
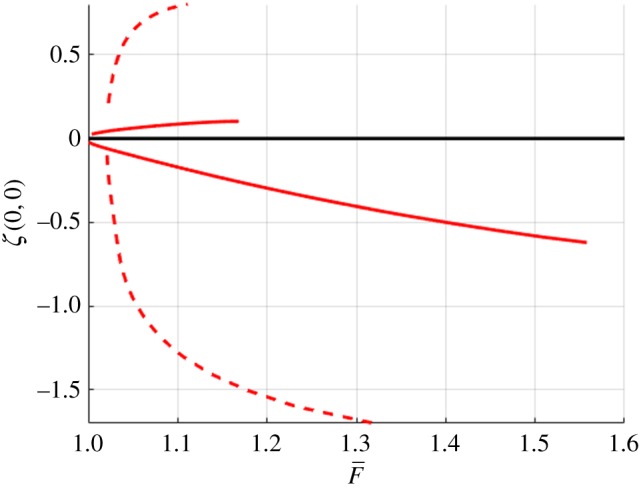
Free surface bifurcation branches of solitary waves with the dashed line representing infinite depth and the solid line is *h* = 1. (Online version in colour.)

Figure 6 shows the comparison of the branches of solutions of solitary waves, normalized by *F*_min_ in each case. The dashed line is for infinite depth and the solid line is for *h* = 1. For infinite depth, it can be shown that in non-dimensional units *F*_min_ ≈ 0.4725 and for *h* = 1, *F*_min_ ≈ 1.0272. These are the values used to normalize the horizontal axis. If these solitary branches bifurcated from *F*_min_, then they would be shown to originate from 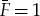 in figure 6. However, this is not the case and the authors believe this is due to numerical errors introduced by truncation or a grid that is too coarse. These numerical difficulties are related to the fact that the solitary waves become more and more oscillatory and less localized as the bifurcation points are approached. Similar difficulties have been encountered previously for gravity–capillary waves (e.g. [42]).

**Figure 7.**
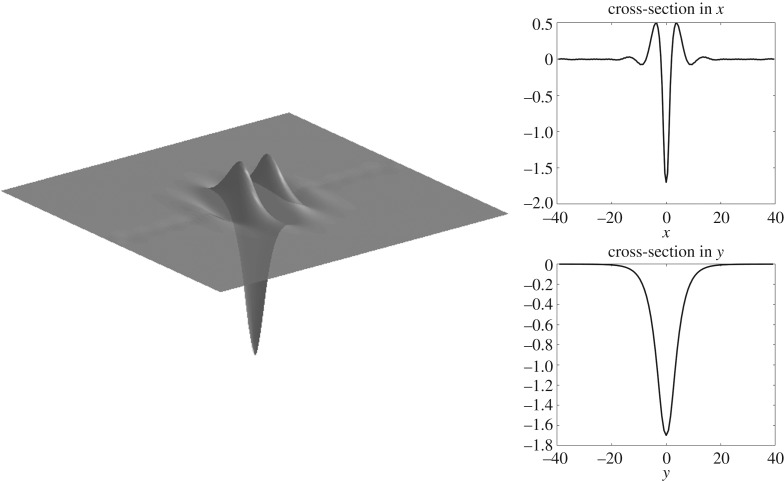
Solitary wave of depression in infinite depth with *F* ≈ 0.52 computed on a quarter of the domain with *n* = 80, *m* = 50, Δ*x* = 0.5, Δ*y* = 0.8, but shown on the full domain.

**Figure 8.**
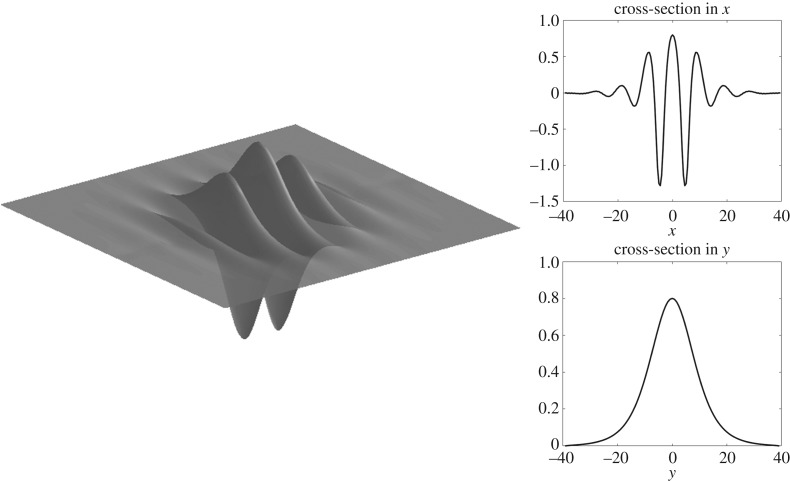
Solitary wave of elevation in infinite depth with *F* ≈ 0.53 computed on a quarter of the domain with *n* = 80, *m* = 50, Δ*x* = 0.5, Δ*y* = 0.8, but shown on the full domain.

**Figure 9.**
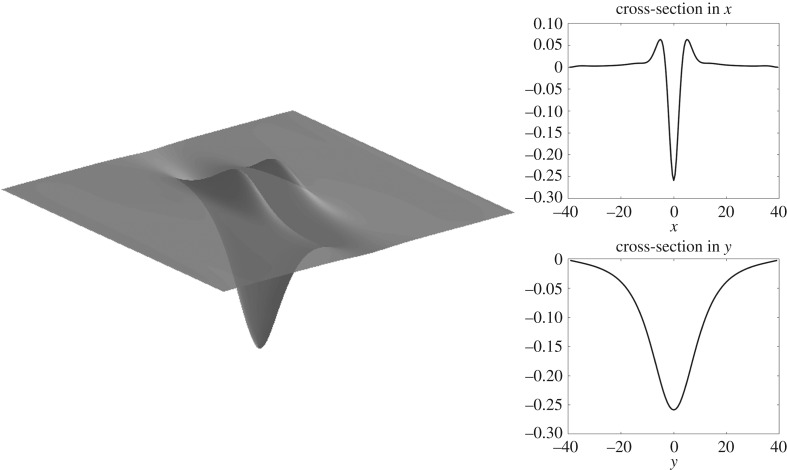
Solitary wave of depression for *h* = 1 with *F* ≈ 1.2 computed on a quarter of the domain with *n* = 80, *m* = 50, Δ*x* = 0.5, Δ*y* = 0.8, but shown on the full domain.

**Figure 10.**
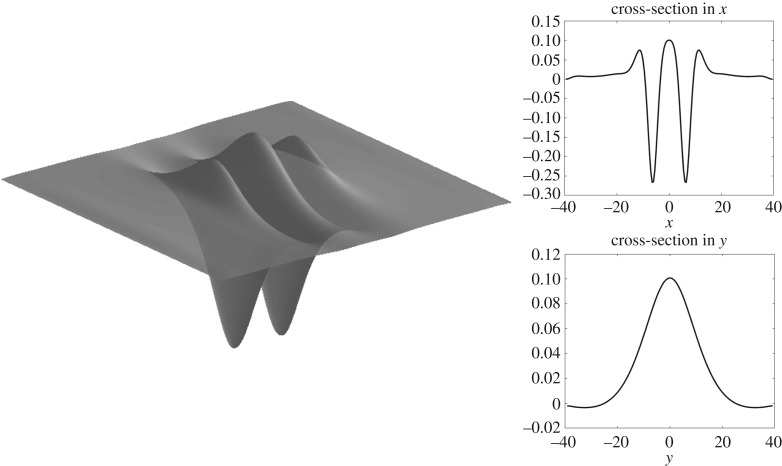
Solitary wave of elevation for *h* = 1 with *F* ≈ 1.2 computed on a quarter of the domain with *n* = 80, *m* = 50, Δ*x* = 0.5, Δ*y* = 0.8, but shown on the full domain.

Figure 6 (taken together with figures 7–10) shows that solitary waves in finite depth have a smaller free surface slope than those for infinite depth for a similar distance (in *F*) from the bifurcation point. This implies that the solitary waves' existence requires smaller nonlinear effects for finite depth than those required for infinite depth. This is numerical evidence that, for *h* = 1, branches of solitary wave solutions bifurcate from zero amplitude, a conclusion that cannot be drawn for waves in infinite depth. This agrees with asymptotic results [36] that suggest that, for infinite depth, these branches do not bifurcate from zero amplitude, whereas in shallower water they do.

It becomes difficult to follow the branches numerically for large-amplitude solutions. In particular, the elevation branch may have turning points as in two dimensions [49], but more grid points are needed for a good accuracy, and this become prohibitive computationally.

Solitary waves are shown in figures 7 and 8 for infinite depth and figures 9 and 10 for *h* = 1. The full wave profiles are shown on the left and the cross-sections on the right. As anticipated from figure 6 with the infinite depth branches having higher amplitude, we see that, in the wave profiles as well, infinite depth waves show more oscillations in the *x*-direction, but finite depth waves are less compact in the *y*-direction. On the elevation branches, the elevation of the wave at the centre point (*x*, *y*) = (0, 0) becomes smaller in magnitude than the depressions which are adjacent to it, similar to the gravity–capillary wave problem [42].

## 5. Conclusion and discussion

In this work, we employ the boundary integral equations method [40,45] to compute three-dimensional solitary flexural–gravity waves for water of finite and infinite depth, covered by an ice sheet, using the model presented by Plotnikov & Toland [11]. This model is very nonlinear with a high number of derivatives, as shown in (2.2), and therefore is numerically difficult to implement. In this case, the usual numerical aids such as iterative methods for matrix inversions and use of inexact Jacobians have not proved to be successful. However, working on a quarter of the domain and using the full symmetry, we are still able to compute the branches of forced and unforced solutions shown in figures 4–6. Sample figures of the wave profiles are also provided, allowing for comparison of solutions in infinite and finite depth as well as depression and elevation waves. These waves are similar to solutions for gravity–capillary waves [43].

One issue is still left to resolve, and that is the asymptotic predictions for the start of the bifurcation branch. It was shown in [36] that flexural–gravity solitary waves in infinite depth bifurcate from a finite-amplitude solution. However, numerically this is difficult to prove. Owing to computational cost, there is a limit to how well these waves can be resolved, resulting in numerical errors due to truncation close to the start of the bifurcation branches, as shown in figure 6, where the branches do not begin at the predicted values of *F*. In shallow water, such as here with *h* = 1, the numerical results suggest that the branch starts from zero amplitude, as predicted by the weakly nonlinear model [36]. There has already been work on using high-performance computing techniques to improve the accuracy of similar computations [46], but more methods need to be developed for nonlinear regimes with higher order derivatives such as the model used for flexural–gravity waves.

It is worth noting that some large-amplitude solutions calculated here may become unphysical, as the strain of the ice plate may be higher than the yield strain of ice. In this case, the elastic model for the sheet will become unrealistic and different models should be used (see [32] for a more detailed discussion).

## Data accessibility

This article has no additional data.
